# CircFAM188A Regulates Autophagy via miR‐670‐3p and ULK1 in Epithelial Ovarian Carcinoma

**DOI:** 10.1002/cnr2.2128

**Published:** 2024-09-04

**Authors:** Min Yong, Yuhua Zeng, Yuqin Yao, Miyuan Yang, Furong Tang, Hongtao Zhu, Jianguo Hu

**Affiliations:** ^1^ Center for Reproductive Medicine, Department of Obstetrics and Gynecology Affiliated Hospital of North Sichuan Medical College Nanchong People's Republic of China; ^2^ Department of Obstetrics and Gynecology Affiliated Hospital of North Sichuan Medical College Nanchong People's Republic of China; ^3^ Department of Clinical Laboratory Affiliated Hospital of North Sichuan Medical College Nanchong People's Republic of China; ^4^ Department of Pediatrics Nanchong Center Hospital Nanchong People's Republic of China; ^5^ Department of Obstetrics and Gynecology Second Affiliated Hospital, Chongqing Medical University Chongqing China

**Keywords:** autophagy, Circ‐FAM188A, epithelial ovarian cancer (EOC), miR‐670‐3p, Unc‐51 like autophagy activating kinase 1 (ULK1)

## Abstract

**Background and Aims:**

CircRNAs and autophagy are closely involved in the physiological and pathological processes of ovarian cancer; however, their exact mechanisms are still undetermined. This investigation aimed to elucidate the function and associated pathways of circFAM188A, which modulates proliferation, autophagy, and invasion in ovarian cancer (EOC).

**Methods:**

The expression of circFAM188A in the tissues of EOC patients was assessed via RT‐PCR. To elucidate proliferation, invasion, and autophagy in the tumor cells, Transwell, 5‐ethynyl‐2′‐deoxyuridine (EdU), and mRFP‐GFP‐LC3 reporter assays were conducted. The binding sites between circ‐FAM188A and the miR‐670‐3p, miR‐670‐3p and YY1 were predicted using bioinformatics and verified by dual‐luciferase reporter assays. Pulldown assays demonstrated binding between ULK1 and circ‐FAM188A. ULK1 was found to be crucial in the initial stage of autophagy. Moreover, an in vivo xenograft model was established by subcutaneous injection of nude mice with EOC cells.

**Result:**

Expression of circ‐FAM188A was increased in EOC tissues relative to normal ovarian tissues and circ‐FAM188A overexpression promoted proliferation, invasion, and autophagy; these effects were reversed by circ‐FAM188A silencing. miR‐670‐3p and circ‐FAM188A co‐localized in the cytoplasm. circ‐FAM188A enhanced YY1 expression by sponging miR‐670‐3p and was also shown to interact with ULK1.

**Conclusion:**

It is thus suggested that circ‐FAM188A modulates autophagy by sponging miR‐670‐3p as well as interacting with ULK1.

## 1 Introduction

Epithelial ovarian cancer (EOC) is a common tumor of the female reproductive system. It is associated with high mortality and substantially threatens women's health. The primary treatment for EOC is comprehensive staging surgery with chemotherapy [1, 2]. An exploration of the pathogenesis and effective EOC treatment targets is thus essential. Associations between circular RNA (circRNA) and EOC tumor development, immunity, and chemotherapy resistance have been reported [3]. For instance, Circ‐0001068 stimulates T‐cell immunity by promoting the expression of PD1 [4]. Furthermore, it has been reported that serum exosomes in cisplatin‐resistant patients have upregulated levels of Circfoxp1 compared with those who are cisplatin‐sensitive. Inhibiting circfoxp1 promotes OC cells' cisplatin sensitivity [5]. Therefore, investigating the role of other cirRNAs in EOC is necessary.

Our previous research identified several circRNAs that were expressed differentially between EOC and normal ovarian tissues using next‐generation transcriptome sequencing. These included circMUC16, which promoted EOC progression by stimulating autophagy via miR‐199a sponging and direct interaction with ATG13 [6]. In another study, after stimulation of autophagy in SKOV3 OC cells by Torin 1, next‐generation sequencing showed variations in circRNA expression, revealing several differentially expressed circRNAs, including circ‐FAM188A. Moreover, circRAB11FIP1 stimulated autophagy in EOC via DSC1 and miR‐129 [7]. Our previous research showed that induction of autophagy by the autophagy inducer Torin 1 markedly increased circ‐FAM188A expression. We thus speculated that circ‐FAM188A might modulate autophagy. Therefore, this investigation aimed to elucidate the role and possible mechanisms by which circ‐FAM188A modulates autophagy in EOC.

## 2 Material and Methods

### 2.1 Tissue Specimens

Sixty‐seven EOC specimens, together with 48 normal tissues, were acquired during surgery and cryopreserved in liquid nitrogen. The present study was approved by the ethical board of the Affiliated Hospital of North Sichuan Medical College. The criteria for inclusion in the study were patients with primary EOC who were treated at the Affiliated Hospital of North Sichuan Medical College between January 2020 and December 2022 and who had not received presurgical chemotherapy, with a pathologically confirmed diagnosis following surgery.

### 2.2 Cell Culture

The human EOC cell lines A2780, ES‐2, SKOV3, and COC1 were cultured at 37°C and 5% CO_2_ in RPMI 1640 media (Sigma‐Aldrich, R8758) augmented with 1% penicillin–streptomycin (Beyotime, C0222) and FBS 10% (04‐00 1‐1A, Biological Industries).

### 2.3 Assessment of Cell Proliferation

The Cell‐Ligh 5‐ethynyl‐2′‐deoxyuridine (EdU) imaging detection kit (Ruibo Biotechnology, China) was used to measure cell proliferation according to the manufacturer's instructions. A2780 and SKOV3 cells were seeded in 24‐well plates and then treated with reagent‐A (50 μM) for 4 h, washed gently with PBS, and fixed with 4% paraformaldehyde (PFA). The cells were then stained with DAPI/Apollo 567 and evaluated and imaged under fluorescence microscopy.

### 2.4 Assessment of Invasion

Matrigel (BD Bioscience, USA) was diluted in RPMI to a ratio of 1:8 and was used to uniformly the upper chamber of a Transwell apparatus (Corning, Lowell, MA, USA) and polymerized at 37°C. Five‐hundred microliters of medium was added to 24‐well plates, and 5 × 10^4^ cells in 200 μL of medium were grown in the Transwell chambers for 48 h, after which the cells that had invaded were fixed with 4% PFA, stained in crystal violet (0.5%) (C0121, Beyotime Institute of Biotechnology), washed with PBS, imaged, and quantified under microscopy.

### 2.5 Quantitative Polymerase Chain Reaction

Total RNA was extracted from the cells using a Total RNA Rapid Extraction Kit (Bioteke, RP1201), according to the provided instructions, and reverse‐transcribed to cDNA using a circRNA fluorescence quantitative detection kit (GS0201, Geneseed, China). The 2^−ΔΔCT^ method was used to quantify gene expression. The primer sequences were as follows:

- Circ‐FAM188A: F: 5′‐AATCTCCTGCTGACGGGACA‐3′ and R: 5′‐AGGTGCAATAACAGCACAGGG‐3′.
- miR‐670‐3p:F: 5′‐CTGATCGTGAGGAGAGTGT‐3′ and R: 5′‐GGTCTTCGACATCGGGGCGG‐3′.

### 2.6 Dual‐Luciferase Reporter Gene Assays

SKOV3 and A2780 cells were grown in 24‐well plates and then transfected for 24 h with control or 100 nM miR‐670‐3p mimics, co‐transfected with specific vectors (0.8 μg) (Yin Yang1 [YY1]‐mut or WT and circ‐FAM188A‐mut or WT) together with Renilla plasmids (1 μg). The relative firefly and Renilla luciferase activities were assessed according to the instructions of the Dual‐Luciferase Reporter Assay System (Promega, E1910) [8].

### 2.7 Western Blotting (WB)

Total proteins were extracted after cell lysis with RIPA buffer (P0013B, Beyotime) containing PMSF (1%, ST506, Beyotime). The proteins were quantified, denatured in a boiling‐water bath, separated on SDS‐PAGE, and transferred to PVDF membranes. The blots were blocked with 5% milk for 2 h at room temperature and then incubated overnight at 4°C with primary antibodies rabbit anti‐ULK1 (ab177472, Abcam), anti‐YY1 (Cat no. 63227, CST), anti‐LC3I/II (Cat no. L7543, Sigma Aldrich), anti‐P62 (Cat no. #23214, CST) and anti‐GAPDH (Cat no. 10494‐1‐AP, Proteintech). The blots were then washed thoroughly and incubated with a secondary antibody for 2 h at ambient temperature, rinsed again, and visualized using ECL Luminescent Solution.

### 2.8 RNA Immunoprecipitation

Magna RNA immunoprecipitation (RIP) (Millipore, USA) was used for RIP, according to the provided directions. Total RNA was extracted from SKOV3 cells by lysis buffer. Magnetic beads were treated at 4°C with anti‐ULK1 and negative control IgG antibody (Abcam, UK), incubated with the cell lysates for 3 h, and rinsed six times with a cold RIP wash buffer. The isolated RNAs were assessed via qRT‐PCR.

### 2.9 RNA Pulldown/MALDI‐TOF‐MS Analyses

RNA pulldown assays were used to identify proteins that interacted directly with circFAM188A, using a Pierce Magnetic RNA‐Protein Pull‐Down kit (20164, Thermo‐Fisher) [9]. The proteins were separated on 10% SDS‐PAGE gels, which were stained with Fast Silver Stain (P0017S, Beyotime). Matrix‐assisted laser desorption//ionization‐time of flight mass spectrometry (MALDI‐TOF‐MS) was then used to identify selected proteins.

### 2.10 mRFP‐GFP‐LC3 Fluorescence for Tracking Autophagic Flux

The detailed method has been published in the previous article [8]. Lentiviral vectors (LV2, Genepharma) expressing shCircFAM188A or not (circFAM188A‐LV2‐1, circFAM188A‐LV2‐2, or circFAM188A‐LV2‐NC [negative control]) and mRFP‐GFP‐LC3 were transfected into SKOV3 cells. Vectors expressing circFAM188A‐LV6‐NC (negative control), circFAM188A‐LV6 (for circFAM188A overexpression, Genepharma), or mRFPGFP‐LC3 were transfected into A2780 cells. LC3 dot images were acquired by confocal microscopy and counted using Image Pro version 6.0. The knockdown sequences of the vectors were as follows:

- circFAM188A‐LV2‐1: 5′‐GAGTGCTCAGGAATGAGGTTT‐3′;
- circFAM188A‐LV2‐2: 5′‐GTGCTCAGGAATGAGGTTTGT‐3′;
- ATG5: 5′‐CAAUCCCAUCCAGAGUUGCUUGUGA‐3′ [7];
- ULK1‐1: 5′‐AAGGACCGCAUGGACUUUGAU‐3′ [10];
- ULK1‐2: 5′‐GGUACCUCCAGAGCAACAUtt‐3′ [11];
- YY1:5′‐CGACGGUUGUAAUAAGAAGUU‐3′ [12];
- negative control (NC) siRNA: 5′‐UUCUUCGAAGGUGUCACGUTT‐3′.

### 2.11 Fluorescence In Situ Hybridization Analysis

Probes tagged with FAM or Cy3 for detecting miR‐670‐3p and circFAM188A were synthesized by Genepharma. The analysis was performed using a fluorescence in situ hybridization (FISH) kit (F03401, Genepharma). Cells (1 × 10^4^) were seeded into a confocal culture dish and grown overnight under standard conditions. The cells were then dehydrated with anhydrous ethanol, permeabilized with 0.1% Triton X‐100, and exposed to SSC (2X). Immediately following exposure to an ethanol gradient, the cells were incubated with 20 μg/mL denatured probe mixture overnight at 37°C. The nuclei were stained with DAPI after washing with 0.4 × SSC/0.3% Tween 20. Images were acquired via a confocal microscope.

### 2.12 Immunohistochemistry (IHC)

The tissues were fixed with 4% PFA, embedded in paraffin, sectioned, baked overnight at 56°C, dewaxed, and rinsed three times with PBS. Antigen retrieval was performed with citrate sodium and 3% H_2_O_2_ for 10 min, after which the sections were blocked, incubated overnight at 4°C with primary antibodies, rinsed thoroughly with PBS, incubated for 15 min in a reaction magnifier, washed, labeled for 15 min with a second antibody at 37°C, stained with DAB, and visualized via light microscopy.

### 2.13 Xenograft Analysis

The in vivo protocols were approved by the Animal Care and Use Committee and followed institutional guidelines. The effects of circFAM188A on proliferation in vivo were assessed in BALB/c nude mice (aged 4 weeks) by subcutaneous injection of 100 μL of physiological saline containing circFAM188A‐LV2‐NC or circFAM188A‐LV2‐1. One group of mice was given chloroquine (CQ, 50 mg/kg) by intraperitoneal injection for 3 weeks. Transfected SKOV3 cells (5 × 10^6^) were used for trial 1. For trial 2, 100 μL physiological saline + circFAM188A‐LV6‐NC or circFAM188A‐LV6‐transfected A2780 cells (5 × 10^6^) were administered to the mice. After 4 weeks, the mice were euthanized, and the tumors were harvested for measurement and weight assessment.

### 2.14 Statistical Analysis

SPSS v26.0 (IBM Corp., Armonk, NY, USA) was used for all analyses, including *t*‐tests and chi‐square tests. The chi‐square test was used to compare the associations between circFAM188A expression and the clinicopathological variables of ovarian cancer samples.

Data are presented as mean ± standard deviation of three experimental replicates. *p* values <0.05 were considered statistically significant.

## 3 Results

### 3.1 EOC Cells Had Elevated circFAM188A Levels

**FIGURE 1:**
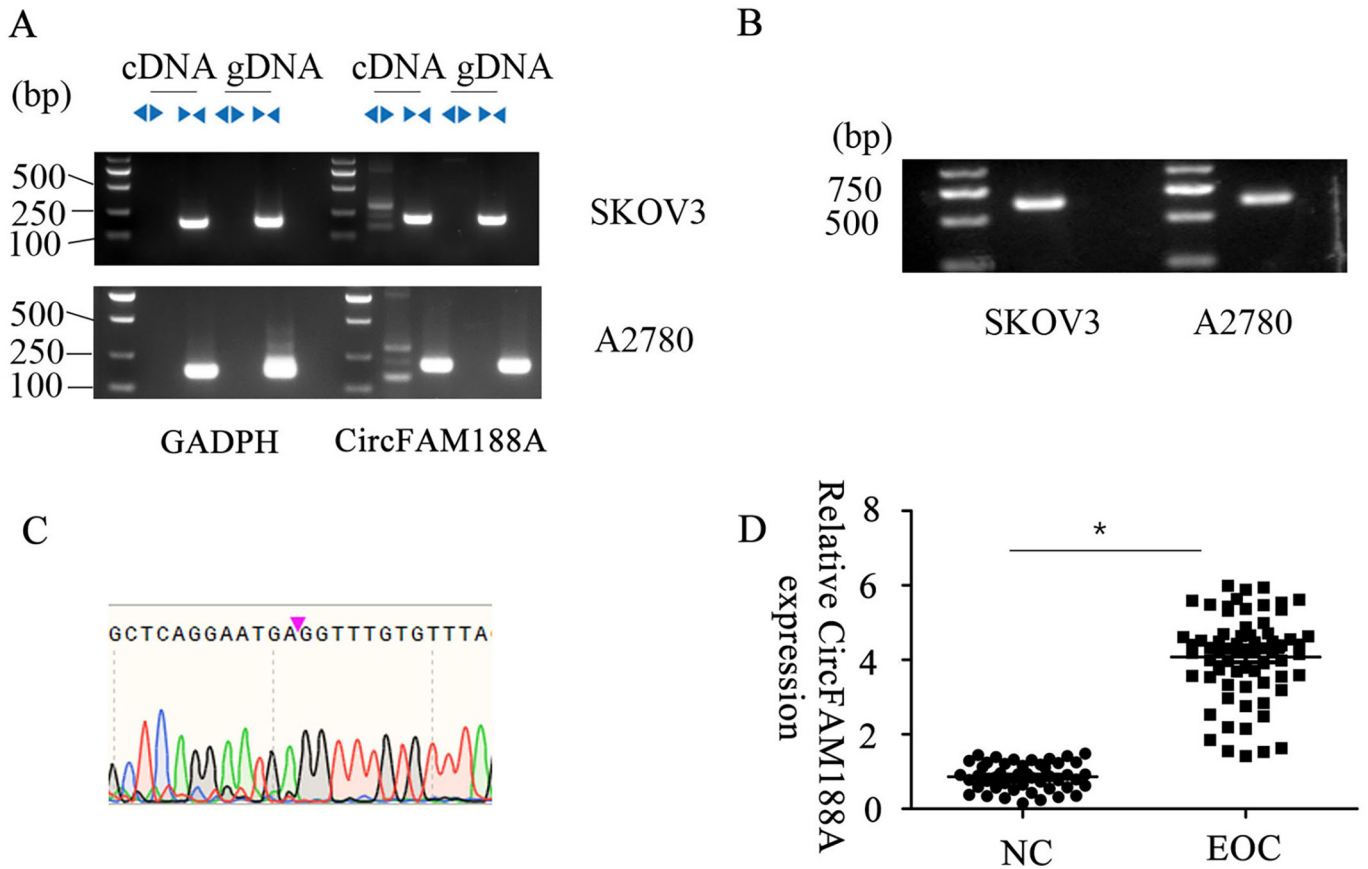
CircFAM188A identification. (A) Agarose gel electrophoresis showing amplification of circFAM188A by a divergent primer within cDNA but not gDNA in A2780 and SKOV3 cells; (B) CircFAM188A trans‐splicing site shown by Sanger sequencing. (C) The length of circFAM188A was 636 bp. (D) The expression of circFAM188A measured by qPCR in 48 normal ovarian tissues and 67 EOC samples. *p < 0.05;

First, the primary circFAM188A features were verified. Using the convergent and divergent primers, circFAM188A was amplified in the gDNA and cDNA of A2780 and SKOV3 cells, which indicated amplification only by the divergent primer in cDNA and not in gDNA and suggesting that trans‐splicing induced circFAM188A cyclization (Figure 1A). Sanger sequencing confirmed the cyclization site (Figure 1C) and revealed that the spliced mature circFAM188A sequence length was 636 kb, amplifiable by a full‐length primer (Figure 1B). CircFAM188A expression was assessed in 67 EOC and 48 normal ovarian tissues, showing that EOC tissues had significantly higher levels of circFAM188A than healthy ovarian tissue (Figure 1D and Table 1).

**TABLE 1:** Association of CircFAM188A expression with clinicopathological characteristics in 67 cases of human epithelial ovarian cancer.

Characteristic	EOC cases	CircFAM188A expression
	*n* = 67	*p* value
Age		
<50	15	<0.05
>50	52	
Serum Ca‐125 level (U/mL)		
<35	13	<0.05
>35	54	
Serum HE4 level (pmol/L)		
<70	17	<0.05
>70	50	
Tumor type		
Clear cell carcinoma	11	<0.05
Serous carcinoma	42	
Mucinous cell carcinoma Serous vs. non‐serous	14	
FIGO stage		
I/II	19	<0.05
III/IV	48	
Grade		
1	14	<0.05
2/3	53	
Tumor size (cm)		
<5		>0.05
>5		
Ascites (mL)		
<100	21	>0.05
>100	46	

### 3.2 CircFAM188A Regulated Autophagy in EOC Tissues

**FIGURE 2:**
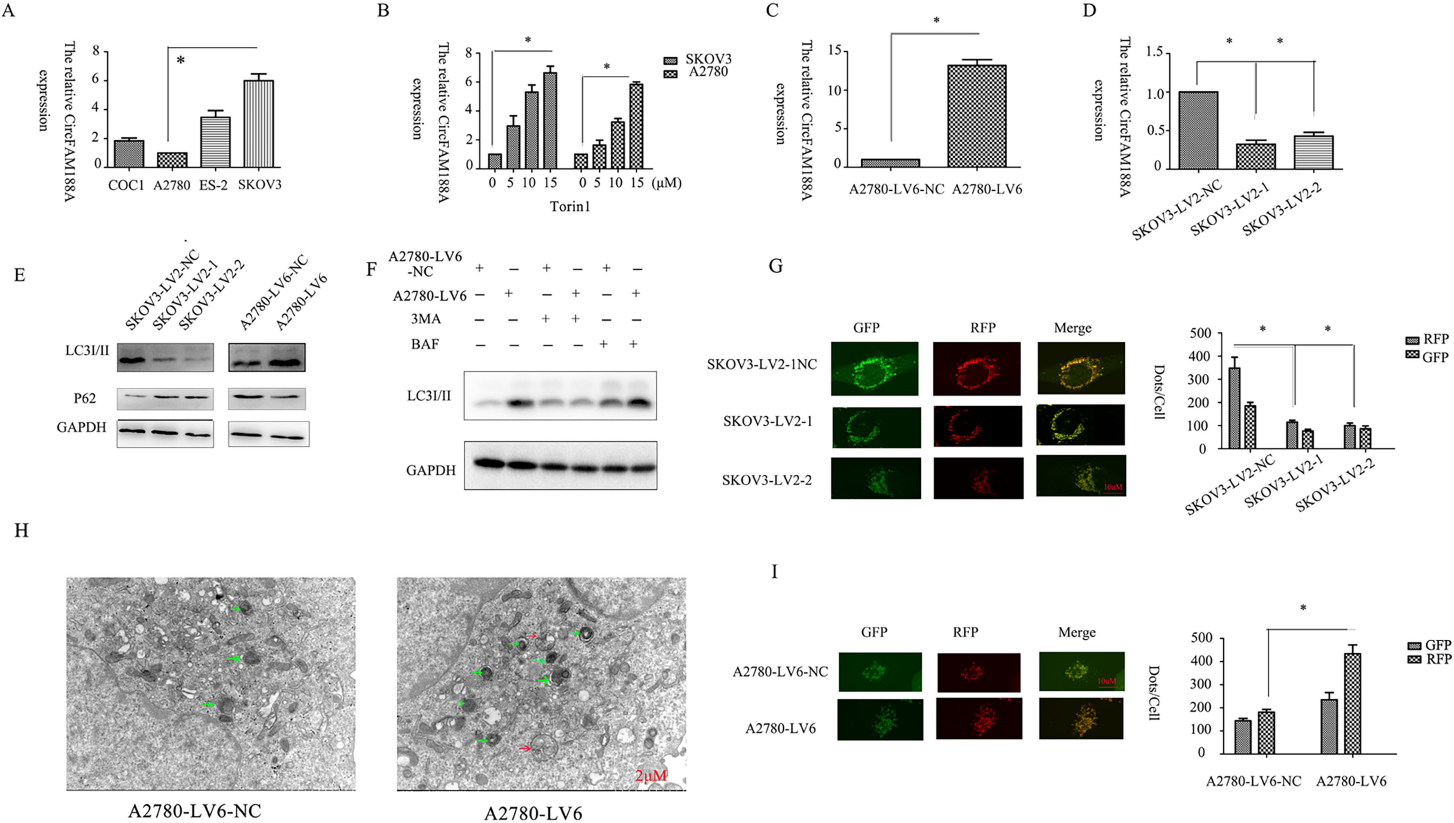
CircFAM188A regulates autophagy. (A) Expression of circFAM188A in COC1, A2780, ES‐2, and SKOV3 cells, measured by qPCR. (B) Expression of circFAM188A after treatment of cells with different concentrations of the autophagy inducer Torin 1, measured by qPCR (*p < 0.05). (C) Overexpression of circFAM188A in A2780 cells, measured by qPCR (circFAM188A overexpression group, A2780‐LV6; negative control group, A2780‐LV6‐NC; *p < 0.05). (D) Efficiency of circFAM188A silencing in SKOV3 cells, measured by qPCR (circFAM188A silencing group 1, SKOV3‐LV2‐1; group 2, SKOV3‐LV2‐2; negative control group, SKOV3‐LV2‐NC; *p < 0.05). (E) LC3I/II and P62 levels shown by western blotting after circFAM188A silencing or overexpression. (F) LC3I/II expression after treatment with 3‐MA or Baf autophagy blockers in the circFAM188A overexpression group (A2780‐LV6) and negative control group (A2780‐LV6‐NC). (G) Autophagic flux shown by confocal microscopy by monitoring mRFP‐GFP‐LC3 fluorescence after circFAM188A silencing; scale bar: 10 μm. (I) Autophagic flux shown by confocal microscopy by monitoring mRFP‐GFP‐LC3 fluorescence after circFAM188A overexpression. *p < 0.05; scale bar: 2 μm. (H) Autophagosomal changes in the circFAM188A overexpression group shown by transmission electron microscopy. *p < 0.05; scale bar: 2 μm.

The circFAM188A levels in EOC cell lines were assessed by quantitative polymerase chain reaction (qPCR), indicating high expression in SKOV3 cells and low levels in A2780 cells. Therefore, SKOV3 cells were used for silencing circFAM188A, while A2780 cells were used for overexpression (Figure 2A). For the assessment of autophagy [7], circFAM188A expression was assessed after 24 h treatment with different doses of the autophagy inducer Torin 1. The results indicated a dose‐dependent increase in circFAM188A in SKOV3 and A2780 cells, suggesting that circFAM188A might be closely linked with autophagy (Figure 2B). CircFAM188A levels were found to be downregulated in SKOV3 cells after knockdown and upregulated in A2780 cells after overexpression (Figure 2C,D). Furthermore, circFAM188A might be associated with autophagy. The results indicated that circFAM188A (SKOV3‐LV2‐1 and SKOV3‐LV2‐2) downregulation suppressed the levels of LC3 while promoting P62, whereas circFAM188A (A2780‐LV6) overexpression had the opposite effect (Figure 2E). To elucidate why LC3 aggregation occurred with circFAM188A overexpression, the autophagy inhibitors 3‐bafilomycin A1 (Baf A1) and methyladenine (3MA) were used to promote the formation and maturation of autophagosomes. The results indicated that 3MA reversed the increase in LC3 due to circFAM188A upregulation, while Baf A1 further increased LC3. Therefore, the upregulation of circFAM188A increased LC3 by stimulating autophagy (Figure 2F). Autophagic flux was tracked using the autophagy double‐labeled adenovirus (mRFP‐GFP‐LC3) [13]. CircFAM188A overexpression increased mRFP dots (Figure 2I), whereas circFAM188A knockdown reduced mRFP dots (Figure 2G). Changes in the autophagosomes were further confirmed by transmission electron microscopy (Figure 2H). Therefore, circFAM188A overexpression stimulates autophagy.

### 3.3 CircFAM188A Regulated Proliferation and Invasion in EOC Cells

**FIGURE 3:**
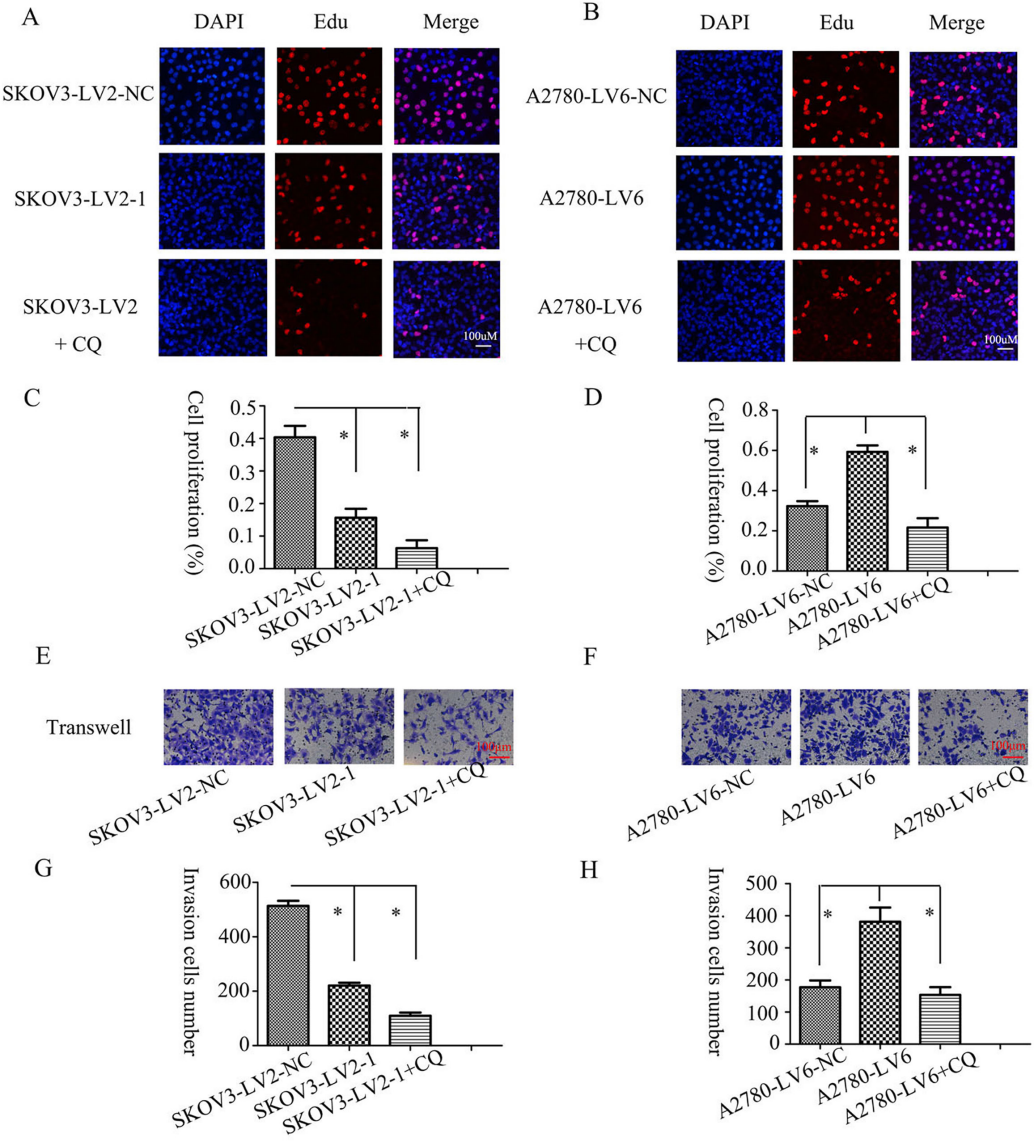
CircFAM188A regulates proliferation and invasion in EOC cells. (A) and (C) EdU assays showing proliferation after circFAM188A silencing or after silencing circFAM188A and treatment with the autophagy blocker CQ; *p < 0.05. (B) and (D) EdU assays showing proliferation after circFAM188A overexpression alone or together with the autophagy blocker CQ; *p < 0.05. (E) and (G) Transwell invasion assays after circFAM188A silencing alone or together with the CQ autophagy blocker; *p < 0.05. (F) and (H) Transwell invasion assays after circFAM188A overexpression alone or with the CQ autophagy blocker; *p < 0.05.

The EOC tissues showed increased levels of circFAM188A, suggesting that it might affect EOC progression. It was found that knockdown of circFAM188A reduced both growth and invasion in SKOV3 cells (Figure 3A,C,E,G), whereas circFAM188A overexpression enhanced both growth and invasion in A2780 cells (Figure 3B,D,F,H). The autophagy inhibitor chloroquine (CQ) further increased the suppression induced by circFAM188A silencing (Figure 3A,C,E,G) and reduced the effects of circFAM188A overexpression (Figure 3B,D,F,H). This suggests that autophagy may promote tumorigenesis in EOC.

### 3.4 CircFAM188A Sponging of miR‐670‐3p

**FIGURE 4:**
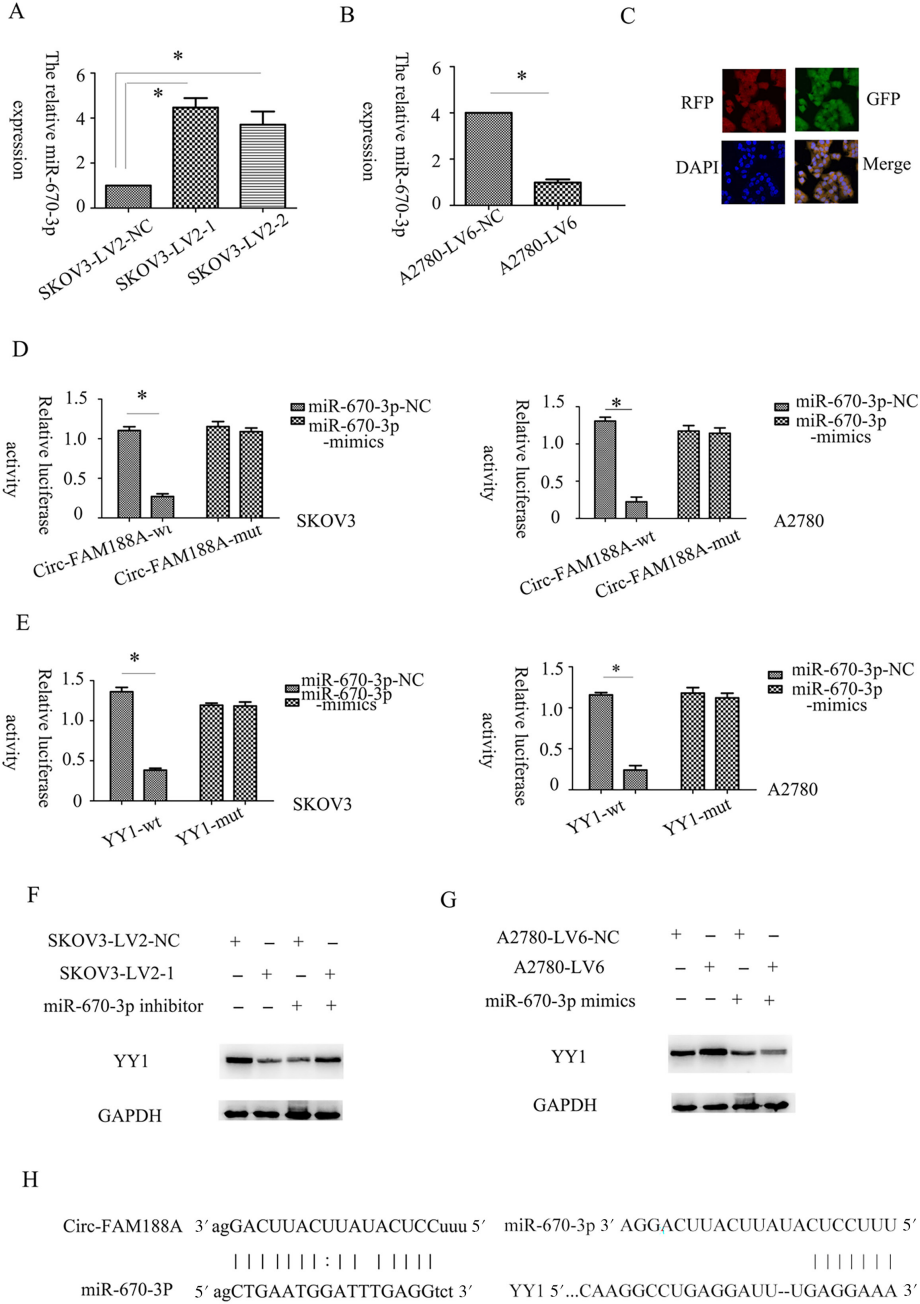
CircFAM188A modulates the miR‐670‐3p/YY1 axis. (A) miR‐670‐3p expression after circFAM188A silencing, shown by qPCR; *p < 0.05). (B) miR‐670‐3p expression after circFAM188A overexpression, shown by qPCR; *p < 0.05). (C) Co‐localization of red fluorescence‐labeled circFAM188A and green fluorescence‐labeled miR‐670‐3p was assessed via the FISH assay. (D) SKOV3 or A2780 cells were transfected with negative control (NC) or miR‐670‐3P mimics, then co‐transfected with circFAM188A‐mut or WT, comprising Renilla and firefly luciferase to assess the luciferase activity, respectively. Error bars indicate standard error; *p < 0.05). (E) SKOV3 or A2780 cells were transfected with negative control (NC) or miR‐670‐3P mimics, then co‐transfected with YY1‐WT or mut, comprising Renilla and firefly luciferase to assess the luciferase activity, respectively. Error bars indicate standard error; *p < 0.05. (F) Western blots showing YY1 expression in each group (SKOV3‐LV2‐NC, SKOV3‐LV2‐1, SKOV3‐LV2‐NC + miR‐670‐3P inhibitor, SKOV3‐LV2‐1 + miR‐670‐3P inhibitor); (G) Western blots showing YY1 expression in each group (A2780‐LV6‐NC, A2780‐LV6, A2780‐LV6‐NC+ miR‐670‐3P mimics, A2780‐LV6 + miR‐670‐3P mimics). (H) Binding site for miR‐670‐3P with circFAM188A/YY1.

The pathways associated with circFAM188A‐induced autophagy, growth, and invasion were then assessed. Differentially expressed miRNAs were predicted using MiRanda (v3.3a). It was found that circFAM188A silencing increased miR‐670‐3p expression (Figure 4A), while circFAM188A overexpression reduced levels of miR‐670‐3p expression levels (Figure 4B), suggesting that circFAM188A sponged miR‐670‐3p. Moreover, the FISH results indicated that circFAM188A and miR‐670‐3p co‐localized in the cytoplasm (Figure 4C). YY1 is known to regulate autophagy and lysosomal biogenesis [14]. Predictions by TargetScan (v 7.2) suggested that YY1 was a putative miR‐670‐3p target (Figure 4H). This was verified using dual‐luciferase reporter gene assays, showing a direct association between miR‐670‐3p and circFAM188A, together with YY1 (Figure 4D,E). CircFAM188A silencing reduced the levels of YY1, and this effect was reversed by suppressing miR‐670‐3p using specific inhibitors (Figure 4F). Ectopic circFAM188A expression enhanced that of YY1, which was rescued by miR‐670‐3p overexpression using miR‐670‐3p mimics (Figure 4G). Furthermore, miR‐670‐3p inhibition enhanced the expression of YY1, whereas its overexpression reduced YY1. Moreover, inhibition of circFAM188A was found to increase miR‐670‐3p expression but reduce that of YY1, whereas overexpression had the opposite effect (Figure 4F). Taken together, circFAM188A modulates the miR‐670‐3p/YY1 axis.

**FIGURE 5:**
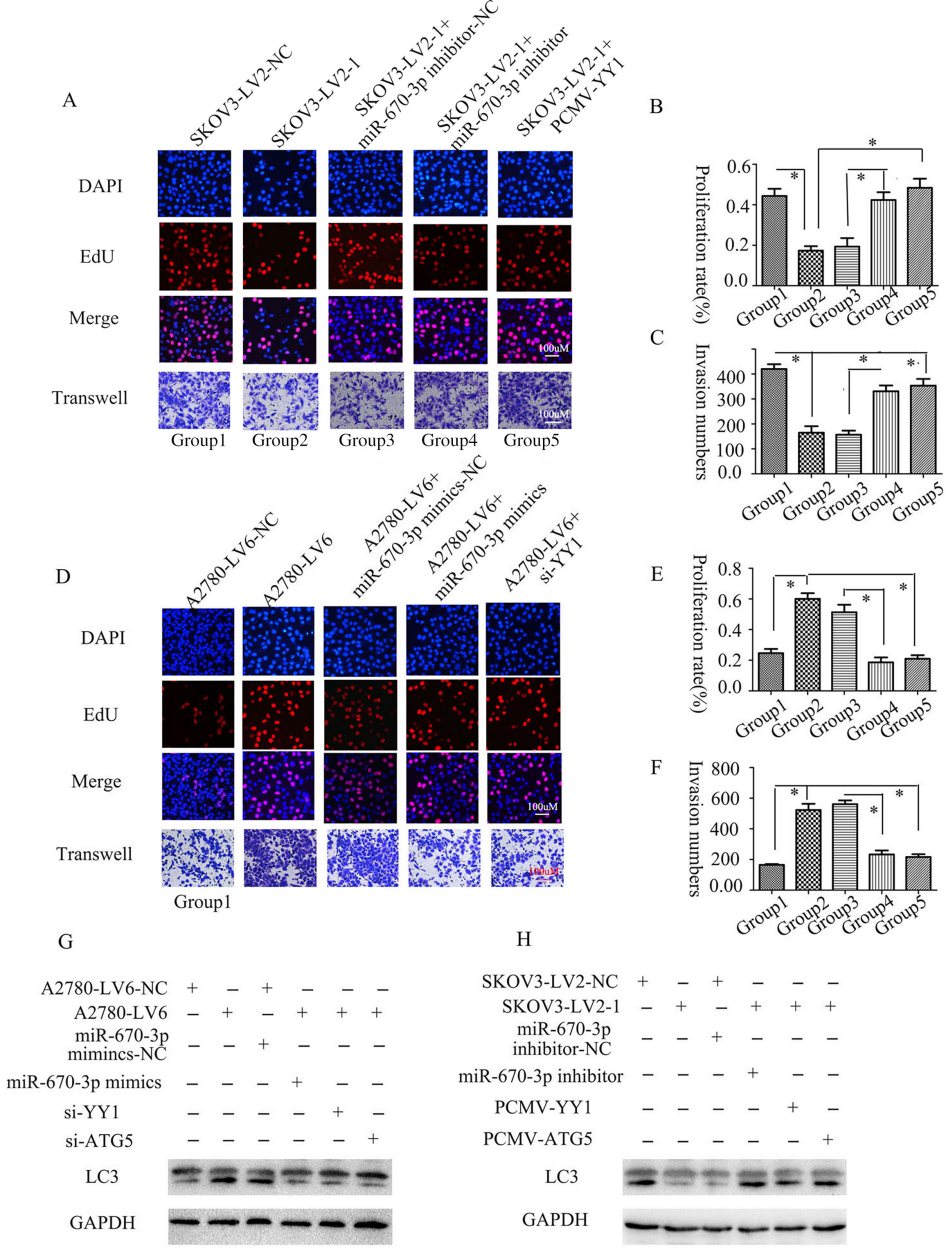
CircFAM188A modulates cellular proliferation, invasiveness and autophagy through the miR‐670‐3p/YY1 axis. (A)–(C) EdU and Transwell assays were used to assessing proliferation and invasion, respectively, in all groups (group 1, SKOV3‐LV2‐NC; group 2, SKOV3‐LV2‐1; group 3, SKOV3‐LV2‐1+ miR‐670‐3P inhibitor‐NC; group 4, SKOV3‐LV2‐1+ miR‐670‐3P inhibitor; group 5, SKOV3‐LV2‐1 + PCMV5‐YY1); Error bars indicate standard error; *p < 0.05. (D)–(F) EdU and Transwell assays were used for assessing proliferation and invasion, respectively, in all groups (group 1, A2780‐LV6‐NC; group 2, A2780‐LV6; group 3, A2780‐LV6+ miR‐670‐3P mimics‐NC; group 4, A2780‐LV6+ miR‐670‐3P mimics; group 5, A2780‐LV6 + si‐YY1). Error bars indicate standard error; *p < 0.05. (G) Western blots showing LC3I/II expression in each trial (trial 1, A2780‐LV6‐NC; trial 2, A2780‐LV6; trial 3, A2780‐LV6+ miR‐670‐3P mimic‐NC; trial 4, A2780‐LV6+ miR‐670‐3P mimics; trial 5, A2780‐LV6 + si‐YY1; trial 6, A2780‐LV6 + si‐AYG5); (H) Western blots showing LC3I/II expression in each trial (trial 1, SKOV3‐LV2‐NC; trial 2, SKOV3‐LV2‐1; trial 3, SKOV3‐LV2‐1+ miR‐670‐3P inhibitor‐NC; trial 4, SKOV3‐LV2‐1+ miR‐670‐3P inhibitor; trial 5, SKOV3‐LV2‐1 + PCMV5‐YY1; trial 6, SKOV3‐LV2‐1 + PCMV5‐ATG5).

Furthermore, it was found that circFAM188A modulated autophagy, growth, and invasion through the miR‐670‐3p/YY1 axis. Downregulation of circFAM188A reduced growth, invasiveness, and LC3 expression, while this was reversed by miR‐670‐3p inhibitors or YY1 overexpression (Figure 5A,B,C,H). Upregulation of circFAM188A promoted growth, invasiveness, and LC3 expression, whereas miR‐670‐3p mimics and YY1 inhibition had the opposite effects (Figure 5D,E,F,G).

### 3.5 CircFAM188A Interacts Directly With ULK1

**FIGURE 6:**
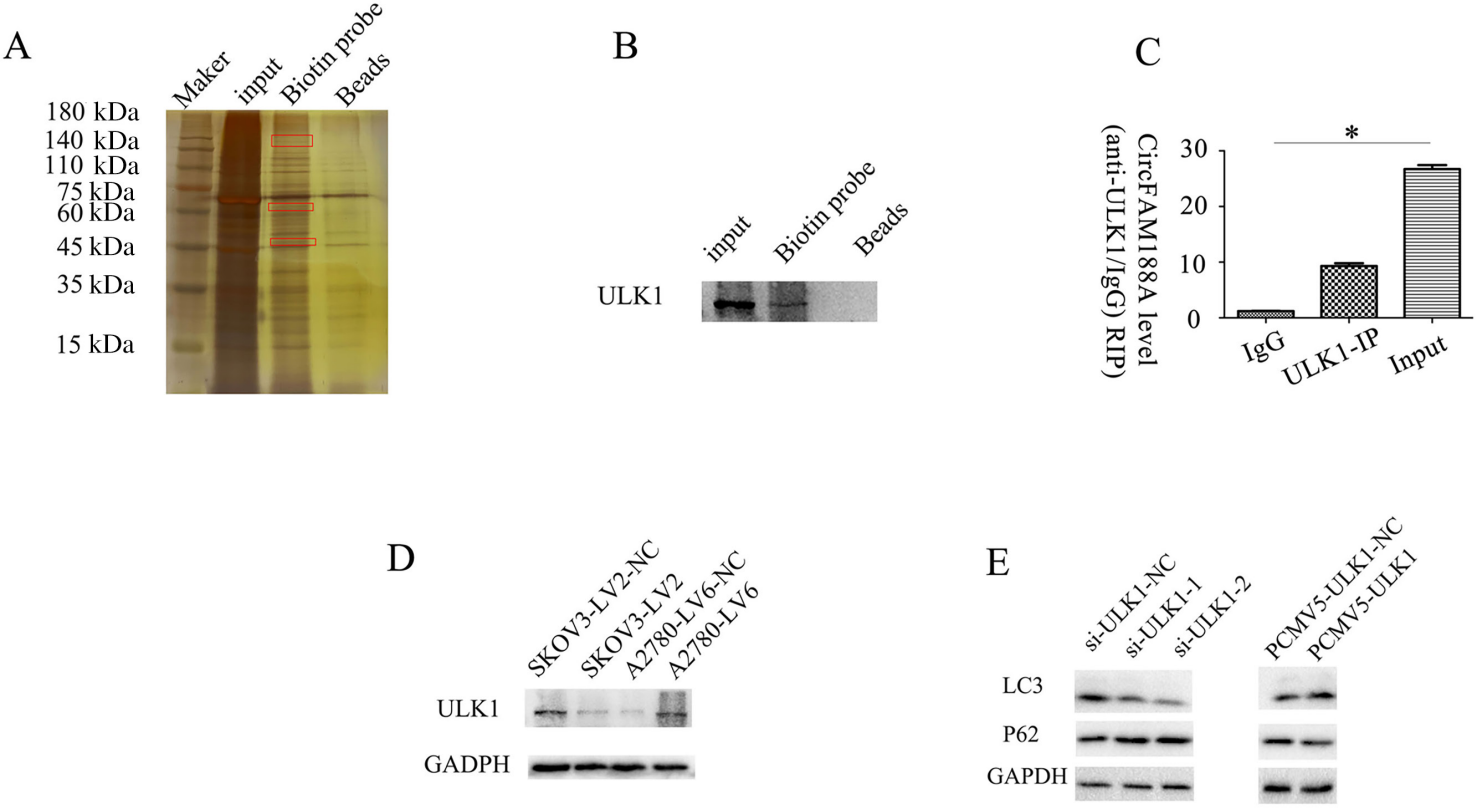
CircFAM188A interacts directly with ULK1. (A) After circFAM188A RNA pulldown, silver staining indicated differential bands (in red box). (B) MALDI‐TOF‐MS results showing ULK1 peptides. Western blotting was used to verify that the circFAM188A biotin‐labeling probe could pull down ULK1. (C) RNA pull‐down assay indicated that ULK1 could be pulled down by circFAM188A. (D) The expression of ULK1 was evaluated by western blotting after silencing or overexpressing circFAM188A. (E) Western blots showing LC3 I/II and P62 expression after ULK1 silencing or over‐expression.

Using a biotin‐labeled CircFAM188A probe, the complexed proteins were pulled down and separated on SDS‐PAGE, as seen in the silver‐stained gels (Figure 6A). Proteins were identified using MALDI‐TOF‐MS. Among the identified proteins was ULK1, which is modulated by circFAM188A (Figure 6B). The interaction of ULK1 with circFAM188A was verified by RIP and qRT‐PCR (Figure 6C). CircFAM188A silencing reduced ULK1, whereas its overexpression had the opposite effect (Figure 6D). Moreover, when ULK1 was inhibited, LC3 expression was downregulated and vice versa (Figure 6E).

### 3.6 Tumor Growth in Xenograft Mice Was Promoted by circFAM188A

**FIGURE 7:**
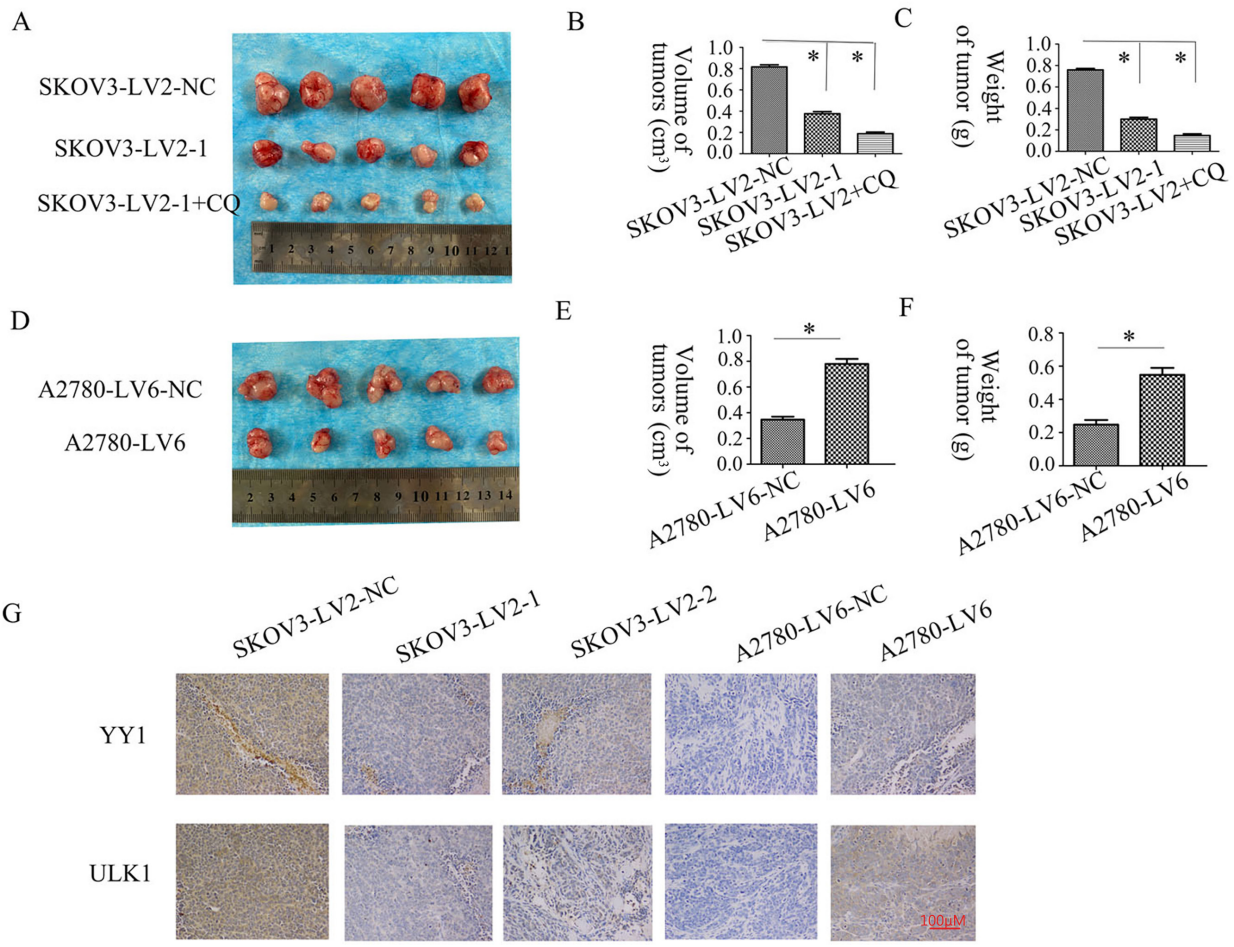
CircFAM188A affects xenograft tumor growth. (A)–(C) Mean volume/weight of xenograft tumors were assessed across all investigations (trial 1, SKOV3‐LV2‐NC; trial 2, SKOV3‐LV2‐1; trial 3, SKOV3‐LV2‐1+ CQ) and (D)–(F) trial 1, A2780‐LV6‐NC; trial 2, A2780‐LV6 (*p < 0.05). (G) Immunohistochemistry showing ULK1/YY1 expression. Original magnification: 200×, scale bar: 100 μm.

The influence of circFAM188A on in vivo tumor proliferation was elucidated. In the circFAM188A‐silencing trial, it was found that both the volume and weight of the tumors were reduced; this effect was enhanced by suppression of circFAM188A using the autophagy inhibitor CQ (Figure 7A,B,C). Tumor weights and volumes were increased after circFAM188A overexpression (Figure 7D,E,F). Furthermore, the levels of YY1 and ULK1 were assessed in the xenograft tumors. Inhibition of circFAM188A reduced these levels, while circFAM188A overexpression had the opposite effect (Figure 7G).

## 4 Discussion

The findings showed increased expression of circFAM188A in tissue samples from EOC patients. CircFAM188A was found to induce autophagy through the miR‐670‐3p/YY1 axis and through interaction with ULK1. It was also shown to promote the growth of xenograft tumors. These findings suggest its potential as a biomarker and potential target for treating EOC.

CircRNAs competitively inhibit transcriptional modulation of mRNA/miRNA [15, 16]. The literature suggests that miR‐670‐3p is a tumor suppressor, as has been shown to reduce glioma growth by targeting ACSL4, essential for ferroptosis [17]. Furthermore, it could also reduce lung adenocarcinoma development and migration. miR‐670‐3p has also been shown to interact with PP7080/UHRF1BP1 to stimulate various biological activities [18], as well as regulate autophagy and growth in cervical cancer. Moreover, the long noncoding RNA ROR1‐AS1 can promote the malignant behavior of these cancer cells. According to mechanistic research, ROR1‐AS1 modulates STC2 by sponging miR‐670‐3p [19].

Additionally, miR‐670‐3p may modulate ovarian cancer. The present study showed that circFAM188A stimulated EOC cell proliferation and invasion. Bioinformatics analysis predicted binding between circFAM188A and miR‐670‐3p, which was subsequently verified by dual‐luciferase reporter gene assays. Therefore, it was speculated that circFAM188A might promote EOC malignancy by interacting directly with miR‐670‐3p.

YY1 is also known to modulate autophagy through transcription factor EB (TFEB) by controlling the expression of genes associated with lysosome biogenesis and autophagy [14]. Moreover, YY1 affects the chemotherapy response of patients with EOC [20]. The present study showed that circFAM188A modulated autophagic flux in EOC cells and that YY1 was a direct target of miR‐670‐3p, suggesting that circFAM188A may modulate autophagy in EOC cells via the miR‐670‐3p/YY1 axis.

CircRNAs can interact with proteins, regulating their stability and altering their location [21]. ULK1 is a serine/threonine protein kinase and the mammalian ortholog of the yeast Atg1. ULK1 regulates autophagosome formation, a key event in autophagy, which has been found to be inhibited by ULK1 knockdown [22]. The literature suggests that increased ULK1 mRNA expression is associated with reduced survival rates in patients with stage III and IV EOC. ULK1 knockdown has been found to elevate carboplatin sensitivity in OC cell lines [23]. The findings of the present study indicated that a biotin‐tagged circFAM188A probe could pull down ULK1. Knockdown of circFAM188A reduced the expression of ULK1, whereas overexpression reversed this effect. Furthermore, circFAM188A upregulation stimulated autophagosome formation and autophagic flux, possibly linked with ULK1, indicating that circFAM188A regulated invasion, autophagy, and proliferation of EOC cells via ULK1.

## 5 Conclusion

Overall, circFAM188A was found to modulate invasion, autophagy, and proliferation in EOC, acting via the miR‐670‐3p/YY1 axis and ULK1 interaction. Therefore, circFAM188A might serve as an effective therapeutic target for EOC.

## Author Contributions

Yuqin Yao, Miyuan Yang and Min Yong conducted animal experiments. Jianguo Hu, Hongtao Zhu and Yuhua Zeng proofread the article. Yuqin Yao and and Furong Tang conducted the western blot and IHC. All authors read and approved the final manuscript.

## Ethics Statement

This investigation follows the Declaration of Helsinki. Informed consent was acquired from all the patients. The Ethics Committee of the Affiliated Hospital of North Sichuan Medical College authorized the study (2021ER(A):051) and approved the animal experiments based on the Animals Use and Care guidelines (North Sichuan Medical College, China I ACUC Issue No. 24).

## Consent

The authors confirm that the tumor burden did not exceed the recommended dimensions and that animals were anesthetized and sacrificed using acceptable methods.

## Conflicts of Interest

The authors declare no conflicts of interest.

## Data Availability

Data sharing is not applicable to this article, as no new data were created or analyzed in this study.
